# Comparison of the Effect of Four Different Abutment Screw Torques on Screw Loosening in Single Implant-Supported Prosthesis after the Application of Mechanical Loading

**DOI:** 10.1155/2021/3595064

**Published:** 2021-07-19

**Authors:** Behnaz Ebadian, Amirhossein Fathi, Saba Khodadad

**Affiliations:** ^1^Dental Implants Research Center, Dental Research Institue, Department of Prosthodontics, School of Dentistry, Isfahan University of Medical Sciences, Isfahan, Iran; ^2^Dental Material Research Center, Dental Research Institue, Department of Prosthodontics, Isfahan University of Medical Sciences, Isfahan, Iran; ^3^Dental Students Research Committee, School of Dentistry, Isfahan University of Medical Sciences, Isfahan, Iran

## Abstract

**Background:**

The complications of implant-supported prostheses can be classified into mechanical and biological ones, one part of which is associated with screw loosening. This study was aimed to compare the effect of four different abutment screw torque techniques on screw loosening in single implant-supported prostheses following the application of mechanical loading.

**Materials and Methods:**

In this experimental study, a total of 40 implants in acrylic blocks (6 × 10 × 20 mm) were mounted perpendicular to the surface. They were then randomly divided into four groups: (1) torquing once with 30 Ncm, (2) torquing three times with 30 Ncm and 5-minute intervals, (3) torquing once with 30 Ncm, opening the screw, and retorquing with 30 Ncm, and (4) torquing once with 35 Ncm. The torque values were confirmed by using a digital torque meter. Then, the samples underwent a force (2 cps, 0.453–11.793 kg) for three hours before the measurement of detorque values. The screw loosening force (torque) was then measured and recorded. The obtained data were analyzed by SPSS (version 22) software using one-way ANOVA and Tukey post hoc test at a 5% error level.

**Results:**

The maximum mean detorque values of the abutment screws in single implant-supported prostheses were reported for groups 4 (27.8 ± 1.3), 1 (26.8 ± 1.3), and 3 (25.1 ± 1.3), and the minimum mean detorque value was found in group 2 (24.9 ± 1.2). Moreover, no significant difference was observed between groups 2 and 3 (*p* > 0.05), but a significant difference was found between groups 1 and 3 and other groups (*p* < 0.05).

**Conclusion:**

The increase in the torque value increased the torque loss. However, the detorque value in group 4 showed the least difference with the value recommended by the manufacturer (30 Ncm).

## 1. Introduction

Since the end of the last century, the dramatic development of dental implant technology has opened up a new era in dental prosthetics [1]. The implant-supported prosthesis is a clinically predictable and beneficial treatment of choice for replacing a lost tooth, but it is not void of the risk of biological, technical, and aesthetic complications [2]. Compared with conventional restorations, the introduction of implant technology has specifically improved the functional and aesthetic results of final restorations, although implant restorations are not ideal [1]. The main biomechanical difference between teeth and implants is the lack of a PDL in implants. The excessive force during centric or excursive movements increases stress on the restoration, creating mechanical complications, such as porcelain chipping, screw loosening or fracture, and, in extreme cases, implant fracture [3].

There are several kinds of implants including endosteal/endosseous, subperiosteal, and intramucosal implants. An endosteal implant contains a blade, screw, pin, or vent that is inserted into the jaw bone by the alveolar or basal bone. Although it is more functional and reliable than the spiral concept, there are certain problems which still have not been overcome. Subperiosteal implants are custom-made frames. They are a metal framework consisting of multiple posts which come out of the gum tissues and are put on the remaining bone. This kind of implant is used in cases that have an insufficient bone or badly atrophied bone. Intramucosal implants are inserted into the oral mucosa. The mucosa is used as an attachment site for the metal inserts [4, 5].

There are two kinds of implant-abutment connection including internal or external, depending on if a geometric characteristic extends above the coronal surface or below. In the internal cone connection-type implant system, the tightening torque is driven by not only the screw height but also the wedge effect due to the conical abutment sinking, and the load is mainly supported by the internal slope of the fixture. Therefore, there is less stress in the abutment screws as compared to the external butt joint. The cold welding inherent to this system favors the torque gain as the friction between the internal implant surfaces and the conical abutment, which makes high stability [6–8].

Besides surgical traumas, high cost and long treatment period have caused clinical problems and mechanical and biological complications for the implant-supported restorations [9–15]. These complications generally include peri-implant mucositis, abutment screw loosening or fracture, abutment or superstructure fracture, crown loosening, and porcelain crack [16–20].

Regarding the mechanical principles of the screw, the application of torque causes elongation and tension, producing a force in the screw known as preload [2]. Preload is also defined as the axial force produced between the threads of the abutment screw and internal parts of the implant in the longitudinal direction [21–24]. Preload should be continued and reduced minimally to prevent the detachment of connections [25]. Preload is positively associated with the screw tightening torque values [1]. An ideal preload is about 60–80% of the yield strength of the materials [26–29]. Only 10% of the torque is converted into preload, while the remaining 90% is used to overcome the friction between connection surfaces [27, 30, 31]. Preload loss has been observed in the first 2-3 minutes [21, 32–34] or 15 hours after tightening [1], even without external forces. The elastic recovery of the screw pulls the assemblies together by applying torque, thereby generating a clamping force [2]. In designing a strong screw connection, the most important functional consideration is the primary clamping force created by screw tightening [30]. The clamping force is usually proportional to the screw tightening torque [30].

The screw is loosened when the external separating force applied to the implant-abutment connection is larger than the clamping force keeping the implant and abutment close together [27, 35]. Bickford [36] divided the screw loosening process into two stages. The initial tensile deformation of the screw is reduced under the influence of the external force, thereby decreasing the clamping force [1]. In the second phase, with more reduction in the clamping force, the micromotion of the implant-abutment interface is intensified, and instability of connections will cause screw loosening [1]. The prevalence of abutment screw loosening has been reported to be 12.7% in the single crowns and 6.7% in the splinted crowns [37].

Despite the high number of clinical and experimental studies, the exact cause of abutment screw loosening has remained unknown [38]. Inadequate tightening torque, improper implant position, inadequate occlusal plane or crown anatomy, racks and coping with poor adaptation, presence of microleakage at the implant-abutment interface, inappropriate design/material of the screw, and heavy occlusal forces can be considered the reasons for abutment screw loosening [17, 39–41]. Abutment screw loosening can cause prosthetic mobility, for which the prosthesis needs to be removed to tighten the abutment [42].

Therefore, the most common complications of abutment screw loosening include gingival inflammation and screw fracture [43]. Various solutions have been suggested to reduce these problems, including the use of diamond-like carbon coating on the abutment screw, retightening of the abutment screw after initial tightening, and increasing the torque level [29, 44–46].

Considering inadequate evidence about abutment screw torquing rates and methods in single implant-supported prostheses, this study was aimed to investigate the effect of different abutment screw torquing methods on the reduction of torque over different periods after application of mechanical cycling. The null hypothesis was there are no differences between different torquing methods.

## 2. Materials and Methods

A total of 40 implants (Zimmer SwissPlus, Implant System. OPB12 platform 4, 8, made in the USA) in acrylic blocks (6 × 10 × 20 mm) were mounted perpendicular to the surface. The perpendicular position of the implant in the resin blocks was confirmed by a dental surveyor (Ney Surveyor, Ney Dental, Bloomfield, CT, USA).

The 40 abutments (OPR, Zimmer, SwissPlus, Carlsbad, USA) were randomly divided into four groups. The abutments in each group were screwed into the implants according to the following conditions:

  Group 1: one time 30 Ncm torque
  Group 2: three times 30 Ncm torques with five-minute intervals
  Group 3: one time 30 Ncm torque, opening the screw, and 30 Ncm retorquing
  Group 4: one time 35 Ncm torque

**Figure 1:**
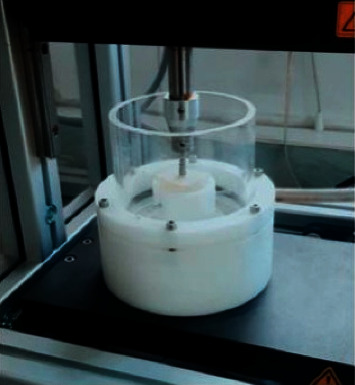
Device for making constant force.

The torque values were confirmed by using a CEDAR digital torque meter (model DID-4, Imada Inc., Northbrook, IL, USA). Then, all samples underwent a force (2 cps, 0.453–11.793 kg) for three hours before the measurement of detorque values (Figure 1). Mechanical cycling was performed by using a servohydraulic testing machine (MTS System Corporation, Eden Prairie, Minnesota, USA) under 4 cycles/s and a force of 31.2 kg. The force was directly applied to the abutment head. Then, the screw loosening force (detorque) was measured and recorded.

(1) $$\begin{array}{c} N=\frac{{\left(Z_{1-\left(\alpha /2\right)+}Z_{1-\beta }\right)}^{2}\left(\sigma_{1}{}^{2}+\sigma_{2}{}^{2}\right)}{d^{2}},\\ \text{significance level }\alpha =\frac{0}{05}⟶Z_{1-\alpha /2}=\frac{1}{96},\\ \text{test power }1-\beta =\frac{0}{80}⟶Z_{1-\beta }=\frac{0}{84},\\ \sigma_{1},\sigma_{2}≃1,\\ d=\frac{1}{25},\\ 10=\frac{{\left(1.96+0.84\right)}^{2}\left(1^{2}+1^{2}\right)}{1.25^{2}}. \end{array}$$

## 3. Results

The results of the Kolmogorov–Smirnov test showed that the abutment screw detorquing values in single implant-supported prostheses in all four groups followed a normal distribution. Furthermore, Levene's test indicated that the variance of abutment screw detorquing values in single implant-supported prostheses was homogenous in all four groups. Therefore, the one-way ANOVA test was run to compare the mean abutment screw detorquing between the study groups (Table 1).

The results of the one-way ANOVA test showed a significant difference among the four study groups in the mean abutment screw detorquing value in single implant-supported prostheses (*p* < 0.001). Moreover, the results of the Tukey post hoc test indicated that the mean abutment screw detorquing value in single implant-supported prostheses was significantly lower in groups 2 and 3 than in group 1 and was significantly lower in group 1 than in group 4 (*p* < 0.05), but there was no significant difference between groups 2 and 3 in this regard (*p* > 0.05) (Table 2).

The results of one-way ANOVA showed a significant difference in the mean percentage of torque loss among the study groups (*p* < 0.001) (Table 3). Furthermore, the Tukey post hoc test indicated that the mean percentage of torque loss was significantly higher in group 4 than in groups 2 and 3 and was significantly higher in groups 2 and 3 than in group 1 (*p* < 0.05), but this difference was not significant between groups 2 and 3 (*p* > 0.05) (Table 4).

## 4. Discussion

The results of this study showed that there are significant differences between different torquing methods, so the null hypothesis was rejected. Abutment screw loosening is caused by improper torque, screw deformation, surface shrinkage, and preload loss due to the implant-abutment tightening torque, which is increased by elevated tightening torque and reduced friction coefficient of the screw. When tightening torque is applied to the abutment screw and external force is applied to the implant superstructure, the compressive force causes the abrasion of surface irregularities and decreases the settling effect preload, thereby loosening the abutment screw [35].

To overcome the reduced clinical preload, a torque similar to the primary torque is suggested to be applied 10 minutes later [47]. In the present study, to compare the effect of four different abutment screw torquing methods on screw loosening in single implant-supported prostheses, retorquing was performed only in two groups after the application of mechanical cycling, which decreased the mean abutment screw detorque compared to the other two groups. This showed a lower torque value in the group with three 30 Ncm torques and five-minute intervals.

The complications of implant-supported prostheses can be classified into mechanical and biological ones, one important part of which is related to the abutment screw loosening [47]. Furthermore, the external forces always lead to transient dynamic deformations at the screw joint. Misfits or deformations present in the implant assemblies increase the abutment screw loosening. A weak interface between implant assemblies increases the initial displacement and causes abrasion in the connection areas, which in turn elevates the distance at the screw connection point. The application of force to the implant assemblies probably leads to tension in both screws and contributes to screw loosening [47]. It has been suggested that conical abutment led to less mechanical complications such as screw loosening or fractures and higher torque preservation. The damage has been observed in the threads of the abutment screws, before and after loading in internal and external connections. The less microleakage in the internal implant was shown in dynamic loading conditions [48].

Conical hybrid connections showed better screw stability than an internal hex connection. A common problem associated with the prosthetic application of dental implants is the loosening and fracturing of screws that hold the prosthesis to the implant which is induced by way of insufficient tightening torque, vibrating micromovement, inappropriate implant position, inadequate occlusal design or crown anatomy, a variant of hex dimension, etc. In addition, factors that affect abutment screws also include component fit, hex height (or depth), and platform diameter. The diameter of the screw may affect the amount of preload applied to the system before deformation. The greater the diameter, the higher the preload that may be applied and the greater the clamping force on the screw joint [6].

In the present study, abutment screw detorque in implant-supported prostheses was investigated in four groups. The maximum value was reported for the group with one 35 Ncm abutment screw torque plus application of mechanical cycling, followed by the group with one 30 Ncm abutment screw torque plus mechanical cycling. The minimum value was found for the group with three 30 Ncm abutment screw torques at five-minute intervals and the application of mechanical cycling. An important point about the values obtained is the number of abutment torques. The minimum mean abutment screw torque in implant-supported prostheses was observed in groups with three torques applied at five-minute intervals. Furthermore, the torque value was lower in the group with two abutment screw torques than the other two groups. Groups 2 and 3 showed no statistically significant difference, which might be because the abutment screw detorque value was equal in both groups.

Pardal-Pardal-Peláez et al. [49] reported that the solutions proposed for the prevention of abutment screw loosening included material selection, screw coating, connection design, and appropriate selection strategies. They also reported that there is a specific strategy to reduce abutment screw loosening [49]. Siamos et al. [29] showed that increasing the frequency of torques greatly reduced the abutment screw detorque force in implant-supported prostheses. Therefore, the results of the present study are in line with those of Pardal-Pardal-Peláez et al. [49] and in contrast to those of Winkler et al. [35, 47].

Researchers have investigated various factors to reduce the abutment screw detorque rate. For example, Lee and Cha [38] studied the abutment screw loosening and torque change relative to the implant screw length and found no significant difference in the torque values among different abutment screw lengths. The torque value required for any screw is introduced by the manufacturer, but it has been shown that it is not stated correctly. Dincer Kose et al. [50] indicated that the amount of preload recommended by the manufacturer was not effective and practical clinically.

The number of cycles needed for abutment screw loosening, especially in the oral cavity, is not known exactly. The presence of biological tissues such as bone, periodontal ligaments, and temporomandibular joint with a different modulus of elasticity has also made this problem more complicated. Moreover, factors associated with the abutment screw affect abutment screw loosening. They also involve yield strength, type of screw, duration of screw use, and its fatigue potential. Hence, the abutment screw loosening potential is variable, and several factors associated with it are still unknown [51]. The abutment screw material can also affect the amount of preload created. Tensile strength and yield strength are higher in the redesigned gold screws than conventional titanium screws. Thus, a higher preload can be created in screws with gold alloy [52].

According to the results of the present study, although the torque loss percentage was higher in group 4 than the other groups, the amount of detorque was close to the amount of fastening torque, which is closer to the torque level recommended by the manufacturer. Considering the application of a mechanical force similar to the welding force, lower screw loosening is expected to occur by application of one 35 Ncm torque to the abutment screw in this implant system. However, future metallurgical studies are required to investigate the mechanical properties of abutment screws under such forces.

The use of only one type of implant-abutment connection and the use of limited mechanical loading, which was done due to economical reasons, were the limitations of our study. In future studies, different types of implant-abutment connection will be checked.

## 5. Conclusion

The maximum mean value of abutment screw detorque was found for one 35 Ncm torque after the application of mechanical cycling. Increasing the number of torques reduced the mean abutment screw detorque.

## Figures and Tables

**Table 1:** Mean value of abutment screw detorquing (Ncm) in single implant-supported prostheses in the study groups.

Group	Mean	SD	*p* value
1	26.8	1.3	<0.001
2	24.9	1.2	
3	25.1	1.3	
4	27.8	1.3	

**Table 2:** Pair comparison of mean values of abutment screw detorquing in single implant-supported prostheses in the study groups.

Groups	*p* value
1 and 2	0.002
1 and 3	0.004
1 and 4	0.045
2 and 3	0.88
2 and 4	<0.001
3 and 4	＜0.001

**Table 3:** Mean percentage of torque loss in the study groups.

Group	Mean	SD	*p* value
1	10.6	4.4	<0.001
2	16.7	3.9	
3	16.4	2.4	
4	20.6	3.6	

**Table 4:** Pair comparison of the mean percentage of torque loss in the study groups.

Groups	*p* value
1 and 2	0.002
1 and 3	0.003
1 and 4	0.004
2 and 3	0.88
2 and 4	0.04
3 and 4	0.03

## Data Availability

The data used to support the findings of this study are available from the corresponding author upon request.
